# Secular Difference in Body Mass Index From 2014 to 2020 in Chinese Older Adults: A Time-Series Cross-Sectional Study

**DOI:** 10.3389/fnut.2022.923539

**Published:** 2022-06-21

**Authors:** Ying Jiang, Xiaomin Zhang, Tianwei Xu, Weiqi Hong, Zhiqi Chen, Xiang Gao, Renying Xu

**Affiliations:** ^1^Department of Clinical Nutrition, Renji Hospital, School of Medicine, Shanghai Jiao Tong University, Shanghai, China; ^2^Department of Psychology, Stockholm University, Stockholm, Sweden; ^3^Caolu Community Health Service Center, Shanghai, China; ^4^Department of Nutrition and Food Hygiene, School of Public Health, Fudan University, Shanghai, China

**Keywords:** body mass index (BMI), the elderly, underweight, overweight, obesity

## Abstract

**Background:**

Body mass index (BMI) is the most widely used parameter to assess the body weight status. Both the increase of BMI (overweight and obesity) and decrease of BMI (underweight) has been associated with high risk of adverse outcome, such as stroke, disability, and even death. However, recent data on secular differences in BMI in the Chinese aged population are limited. The present study provides robust new evidence about the evolving epidemic of obesity among aged adults in China.

**Objective:**

Evaluating secular difference in BMI in a group of Chinese older adults.

**Materials and Methods:**

We analyzed 7 continuous survey years (2014–2020), including 50,192 Chinese aged participants (25,505 men and 24,687 women, aged 71.9 ± 6.1 years, age range: 65–99 years). Information on sex, age, height, and body weight, was collected based on medical history. Participants were classified into four groups: underweight (BMI < 18.5 kg/m^2^), normal weight (18.5 kg/m^2^ ≤ BMI < 25 kg/m^2^), overweight (25 kg/m^2^ ≤ BMI < 30 kg/m^2^), and obesity (BMI ≥ 30 kg/m^2^). Linear regressions were used to assess the secular difference in BMI. Sex and age differences were also evaluated by stratified analyses.

**Results:**

From 2014 to 2020, age-adjusted mean BMI increased by 0.3 kg/m^2^ (95% *CI*: 0.1, 0.5 kg/m^2^) in men, and 0.5 kg/m^2^ (95% *CI*: 0.2, 0.7 kg/m^2^) in women. Age-standardized prevalence of underweight decreased from 3.0 to 2.3% in men, and from 3.0 to 2.1% in women. Age-standardized prevalence of overweight increased in both men (from 40.1 to 41.7%) and women (from 37.8 to 39.8%), and so as obesity (men: from 4.1 to 6.1%; women: from 5.8 to 8.7%).

**Conclusion:**

Our results confirmed that BMI gradually increased from 2014 to 2020. The age-adjusted mean BMI increased by 0.3 kg/m^2^ in older men, and 0.5 kg/m^2^ in older women. The age- and sex-standardized prevalence of overweight and obesity significantly increased, especially in 70–79-year age group, while the prevalence of underweight decreased. The combination of a balanced-diet and physical exercise is needed to maintain optimal BMI range for the aged population.

## Introduction

The number of aged population has dramatically increased in both the developed and the developing countries. Statistics showed that the proportion of older adults (≥ 65 years) in the global population has exceeded 7%, and it is expected to reach 22.59% by the end of this century (1). China, the most populous country in the world, has been experiencing a very fast pace of the population aging process and has the largest older population (2).

The aged population is threatened by both underweight (often referred as malnutrition) and overweight (referred as overweight and obesity). Underweight is much higher among older adults in India, as a recent nationwide community-dwelling survey in India showed that the prevalence of underweight among people aged 65 years and over was 28.4% (3). Underweight in older adults is associated with an increased risk of mortality and morbidity (4, 5), poor self-reported health (6, 7) and physical decline, which have wide ranging acute implications for activities of daily living and quality of life in general (8). Overweight in older adults is associated with a range of chronic diseases, such as cardiovascular diseases, diabetes, some types of cancers, and musculoskeletal disorders (9–15). The constant temptation of making poor health choices like lack of balance in diet, too little exercise and day to day stress are adding to this rising problem worldwide and needs to be addressed.

A Chinese national survey reported that the prevalence of underweight, overweight, and obesity was 5.7, 34.8, and 12.4%, respectively, in population aged 60 years and over, however, they did not report secular change in BMI. Furthermore, data were collected between 2010 and 2013, so it may not reflect BMI changes in the last decade. (16). Moreover, a recent study on 8,244 Korean older persons emphasized the gender differences with men showing higher prevalence of underweight and lower prevalence of overweight/obesity (17), which was not depicted in the recent Chinese studies. Further, not all studies reported the prevalence of underweight. For example, prevalence of underweight has been reported in South Korea (17) and Spain (18), but not in an earlier study in the United States (19), where the prevalence of overweight and obesity was shown to be higher than South Korea and Spain (19).

Body mass index (BMI) is the most widely used parameter to assess body weight status (20). Both the increase of BMI (overweight and obesity) and decrease of BMI (underweight) has associated high risk of adverse outcome, such as stroke, disability, and even death. However, recent data (i.e., after year 2015) on secular differences in BMI or prevalence of obesity in Chinese aged population is limited. To the best of our knowledge, only two studies were identified. One study reported a steady rise in mean BMI and obesity among adults aged 18–69 years from 2004 to 2018, however, with seniors over 70 years old excluded (21). Another study found increasing prevalence of obesity and overweight in Chinese older adults during the period between 2013 and 2018 but did not examine the trend in mean BMI (22).

Therefore, we aimed to evaluate the secular difference in BMI among older people collected from a community-based population annually from 2014 to 2020 comprising a total of 50,192 men and women.

## Materials and Methods

### Study Population

The initial recruitment included 58,947 Chinese aged participants who were from two different community parts: 28,633 participants (17,527 men and 11,106 women, 72.3 ± 6.7 years) were from the Health Management Center, the Renji Hospital, and 30,314 participants (11,880 men and 18,434 women, 72.8 ± 6.8 years) were from the Caolu Community Health Service Center, Shanghai. After excluding participants without information on height and body weight (BW) (*n* = 8,755), a total number of 50,192 participants, whose mean age was 71.9 ± 6.1 years, were finally included in the study (Supplementary Figure 1). Mean BMI was slightly lower for the participants from the Renji Hospital than those from the Caolu Community Health Service Center (24.5 ± 3.2 kg/m^2^ *vs.* 25.2 ± 3.6 kg/m^2^, *p* < 0.01), while the participants from the Renji Hospital is older than those from the Caolu Community Health Service Center (72.1 ± 6.5 years *vs.* 71.8 ± 5.8 years, *p* < 0.01).

### Assessment of Body Mass Index

Body weight and height were measured in light clothes with bare foot. BMI was calculated as weight in kilograms divided by squared height in meters. To facilitate cross-country comparisons, we defined overweight and obesity by the World Health Organization (WHO) criteria (23). Participants were classified into: underweight (BMI < 18.5 kg/m^2^), normal weight (18.5 kg/m^2^ ≤ BMI < 25 kg/m^2^), overweight (25 kg/m^2^ ≤ BMI < 30 kg/m^2^), and obesity (BMI ≥ 30 kg/m^2^).

### Statistical Analysis

In the descriptive statistics, continuous variables were presented as means and standard deviations (SD) while categorical variables were shown as proportion or percentage. We computed age-standardized prevalence of underweight, overweight, and obesity based on the 2020 census of the Shanghai aged population by the direct method (24).

To assess the secular difference in mean BMI where “year” served as the exposure and “BMI” served as the outcome after adjustment of age and sex. As a supplementary, we also analyzed the secular difference of height and body weight, by treating them as separate outcomes. For assessing the prevalence of BMI categories over the time; we further treated prevalence of each BMI category as separate outcomes.

We used linear regressions for all analyses. The *p-*values for trends were determined by linear regression analyses after setting year as a continuous variable.

Men and women were separated in all analyses.

We also showed age-related trends in height, weight, and BMI.

We examined the age-based tendency of BMI, height, and body weight within each individual year in the sensitivity analyses. All statistical analyses were performed with SAS version 9.4 (SAS Institute Inc., Cary, NC, United States). Statistical significance was determined at *p*-value < 0.05.

### Ethics Statement

N/A. As a re-identified analysis, the consent was waived by the Ethics Committee of Renji Hospital, School of Medicine, Shanghai Jiao Tong University.

## Results

### Descriptive Analysis

A total number of 50,192 Chinese aged participants (25,505 men and 24,687 women, mean age: 71.9 ± 6.1 years, age range: 65–99 years) were included in the study, with a mean annual sample of 7,170 individuals. Mean height, body weight, and BMI was 160.5 ± 8.7 cm, 64.2 ± 10.9 kg, and 24.9 ± 3.4 kg/m^2^, respectively (Tables 1A,B). Mean BMI was associated with natural year in women in all age groups, while it was associated with natural year only in 70–79-year-old men (Tables 1A,B).

**TABLE 1A:** General characteristics of 25,505 Chinese aged men from 2014 to 2020.

Age group	Variables	Men (*n* = 25,505)							*p*-value
		2014 (*n* = 2,706)	2015 (*n* = 2,719)	2016 (*n* = 3,226)	2017 (*n* = 5,248)	2018 (*n* = 5,615)	2019 (*n* = 3,244)	2020 (*n* = 2,747)	
65–69 y	Height (cm)	167.1 (6.1)	167.7 (6.0)	168.1 (6.0)	167.8 (6.0)	166.9 (6.7)	167.1 (6.2)	167.6 (6.0)	< 0.01
	BW (kg)	69.8 (9.8)	70.2 (9.5)	70.7 (9.4)	70.4 (10.0)	70.2 (10.1)	70.2 (10.0)	70.5 (9.9)	0.43
	BMI (kg/m^2^)	25.0 (3.0)	24.9 (3.0)	25.0 (3.0)	25.0 (3.1)	25.2 (3.1)	25.1 (3.1)	25.1 (3.1)	0.24
70–79 y	Height (cm)	165.8 (6.1)	166.5 (5.8)	166.6 (6.2)	166.2 (6.1)	165.6 (6.6)	165.5 (6.4)	165.6 (6.3)	< 0.01
	BW (kg)	67.3 (10.0)	67.9 (9.7)	68.3 (9.7)	68.4 (9.8)	68.4 (10.0)	68.7 (10.7)	68.5 (10.5)	0.02
	BMI (kg/m^2^)	24.5 (3.2)	24.5 (3.1)	24.6 (3.2)	24.7 (3.1)	24.9 (3.2)	25.0 (3.3)	25.0 (3.4)	< 0.01
≥ 80 y	Height (cm)	164.7 (5.9)	165.1 (5.7)	164.7 (6.0)	164.9 (6.0)	164.4 (6.7)	163.6 (6.0)	163.5 (5.5)	< 0.01
	BW (kg)	64.9 (10.0)	65.4 (9.8)	64.7 (9.9)	64.6 (9.7)	64.8 (9.9)	65.5 (10.6)	65.6 (10.4)	0.69
	BMI (kg/m^2^)	23.9 (3.4)	24.0 (3.3)	23.8 (3.4)	23.7 (3.3)	24.0 (3.3)	24.4 (3.4)	24.5 (3.5)	0.05
**TABLE 1B |** General characteristics of 24,687 Chinese aged women from 2014 to 2020.									
**Age group**	**Variables**	**Women (*n* = 24,687)**							***p*-value**
		**2014 (*n* = 1,582)**	**2015 (*n* = 1,577)**	**2016 (*n* = 1,964)**	**2017 (*n* = 5,500)**	**2018 (*n* = 5,457)**	**2019 (*n* = 4,527)**	**2020 (*n* = 4,080)**	
65–69 y	Height (cm)	155.1 (5.5)	155.7 (5.8)	156.0 (5.7)	155.7 (5.8)	156.6 (6.4)	155.9 (5.7)	155.9 (5.4)	< 0.01
	BW (kg)	59.8 (9.1)	60.1 (9.3)	59.2 (8.7)	60.5 (9.3)	61.4 (9.7)	60.9 (9.3)	61.1 (9.2)	< 0.01
	BMI (kg/m^2^)	24.8 (3.5)	24.8 (3.4)	24.3 (3.3)	24.9 (3.5)	25.0 (3.5)	25.0 (3.5)	25.1 (3.5)	< 0.01
70–79 y	Height (cm)	153.4 (5.5)	154.0 (5.7)	154.2 (5.6)	153.3 (5.7)	154.0 (6.4)	153.5 (5.9)	153.7 (5.6)	< 0.01
	BW (kg)	58.3 (8.9)	57.4 (8.5)	57.7 (8.7)	58.6 (9.4)	59.8 (9.6)	59.8 (9.8)	59.9 (9.3)	< 0.01
	BMI (kg/m^2^)	24.7 (3.4)	24.2 (3.3)	24.3 (3.3)	24.9 (3.7)	25.2 (3.6)	25.4 (3.8)	25.3 (3.6)	< 0.01
≥ 80 y	Height (cm)	150.5 (5.8)	151.4 (6.3)	151.8 (5.2)	150.6 (6.2)	149.9 (7.1)	150.5 (6.7)	150.7 (6.2)	< 0.01
	BW (kg)	54.2 (8.5)	54.6 (9.2)	55.4 (9.0)	55.5 (9.4)	55.8 (9.4)	56.1 (9.7)	56.1 (9.3)	0.15
	BMI (kg/m^2^)	23.9 (3.4)	23.8 (3.6)	24.0 (3.5)	24.5 (4.0)	24.8 (3.9)	24.7 (3.8)	24.7 (3.7)	< 0.01

*BMI, body mass index; BW, body weight.*

*Data are expressed as means (standard deviations).*

*The difference among the year groups was tested by F test.*

### Age-Specific Trends in Height, Body Weight, and Body Mass Index

Height, body weight, and BMI decreased with age in both men and women (Table 2). For example, compared with men aged 65–69 years (served as reference), men aged 70–79 years lost an average of 1.5 cm (95% *CI*: 1.3, 1.7 cm) in height, 2.0 kg (95% CI: 1.7, 2.3 kg) in body weight and 0.3 kg/m^2^ (95% *CI*: 0.2, 0.4 kg/m^2^) in BMI, and men aged 80 years or more lost an average of 2.8 cm (95% *CI*: 2.6, 3.1 cm) in height, 5.4 kg (95% *CI*: 5.0, 5.8 kg) in body weight and 1.1 kg/m^2^ (95% *CI*: 1.0, 1.2 kg/m^2^) in BMI (Table 2). For subgroup analysis, similar secular differences in height and body weight were confirmed in each individual year (Supplementary Table 1).

**TABLE 2:** Age-specific trends in anthropometric data in 50,192 Chinese aged participants.

Variable	Sex	Age group		
		65–69 y	70–79 y	≥ 80 y
BMI, kg/m^2^	Men	25.0 (25.0, 25.1)	24.8 (24.7, 24.8)	23.9 (23.8, 24.1)
Difference in BMI, kg/m^2^		Ref.	–0.3 (–0.4, –0.2)	–1.1 (–1.2, –1.0)
BMI, kg/m^2^	Women	24.9 (24.9, 25.0)	25.0 (25.0, 25.1)	24.5 (24.4, 24.7)
Difference in BMI, kg/m^2^		Ref.	0.1 (–0.01, 0.2)	–0.4 (–0.5, –0.2)
Height, cm	Men	167.4 (167.3, 167.6)	165.9 (165.8, 166.1)	164.6 (164.4, 164.8)
Difference in height, cm		Ref.	–1.5 (–1.7, –1.3)	–2.8 (–3.1, –2.6)
Height, cm	Women	156.0 (155.9, 156.1)	153.7 (153.6, 153.8)	150.6 (150.4, 150.8)
Difference in height, cm		Ref.	–2.3 (–2.4, –2.1)	–5.4 (–5.6, –5.1)
BW, kg	Men	70.3 (70.1, 70.5)	68.3 (68.1, 68.5)	64.9 (64.6, 65.3)
Difference in BW, kg		Ref.	–2.0 (–2.3, –1.7)	–5.4 (–5.8, –5.0)
BW, kg	Women	60.7 (60.5, 60.9)	59.2 (59.0, 59.4)	55.7 (55.4, 56.0)
Difference in BW, kg		Ref.	–1.5 (–1.8, –1.2)	–5.0 (–5.4, –4.6)

*BMI, body mass index; BW, body weight.*

*The difference between the age groups was tested by linear regression analysis.*

*Data are expressed as means (95% confidence interval).*

### Secular Difference in Mean Body Mass Index

Mean BMI increased with natural year in both men and women after adjustment of age (Table 3). From 2014 to 2020, age-adjusted mean BMI increased by 0.3 kg/m^2^ (95% *CI*: 0.1, 0.5 kg/m^2^) in men, and 0.5 kg/m^2^ (95% *CI*: 0.2, 0.7 kg/m^2^) in women. For subgroup analysis, similar trends were confirmed in women of all age groups, but only in men aged 70–79 years (Supplementary Tables 2A,B).

**TABLE 3:** Trends in anthropometric parameters in 50,192 Chinese aged participants from 2014 to 2020.

Variable	Sex	2014	2015	2016	2017	2018	2019	2020
BMI, kg/m^2^	Men	24.6 (24.5, 24.8)	24.6 (24.5, 24.8)	24.7 (24.6, 24.8)	24.7 (24.6, 24.8)	24.9 (24.8, 25.0)	25.0 (24.9, 25.1)	24.9 (24.8, 25.0)
Difference in BMI, kg/m^2^		Ref	–0.001 (–0.2, 0.2)	0.1 (–0.2, 0.3)	0.1 (–0.1, 0.3)	0.3 (0.1, 0.5)	0.3 (0.1, 0.5)	0.3 (0.1, 0.5)
BMI, kg/m^2^	Women	24.7 (24.5, 24.9)	24.4 (24.2, 24.6)	24.3 (24.1, 24.4)	24.8 (24.8, 25.0)	25.1 (25.0, 25.2)	25.1 (25.0, 25.2)	25.2 (25.0, 25.3)
Difference in BMI, kg/m^2^		Ref	–0.3 (–0.6, 0.04)	–0.4 (–0.7, –0.1)	0.2 (–0.1, 0.4)	0.4 (0.1, 0.6)	0.4 (0.2, 0.7)	0.5 (0.2, 0.7)
Height, cm	Men	166.3 (166.1, 166.5)	166.9 (166.6, 167.1)	167.1 (166.9, 167.3)	166.8 (166.7, 167.0)	166.1 (165.9, 166.2)	166.0 (165.8, 166.2)	166.2 (166.0, 166.5)
Difference in Height, cm		Ref	0.6 (0.1, 1.0)	0.8 (0.4, 1.2)	0.5 (0.2, 0.9)	–0.2 (–0.6, 0.1)	–0.3 (–0.8, 0.1)	–0.1 (–0.5, 0.4)
Height, cm	Women	153.7 (153.4, 154.0)	154.3 (154.0, 154.6)	154.6 (154.3, 154.8)	154.1 (153.9, 154.2)	154.7 (154.5, 154.9)	154.2 (154.0, 154.3)	154.2 (154.1, 154.4)
Difference in Height, cm		Ref	0.6 (0.1, 1.2)	0.9 (0.4, 1.4)	0.4 (–0.05, 0.8)	1.0 (0.6, 1.4)	0.4 (0.02, 0.9)	0.5 (0.1, 1.0)
BW, kg	Men	68.2 (67.8, 68.6)	68.7 (68.3, 69.0)	69.0 (68.6, 69.3)	68.9 (68.6, 69.1)	68.8 (68.5, 69.1)	68.9 (68.5, 69.2)	68.9 (68.6, 69.3)
Difference in BW, kg		Ref	0.4 (–0.2, 1.1)	0.7 (0.1, 1.4)	0.6 (0.05, 1.2)	0.6 (–0.01, 1.2)	0.7 (0.004, 1.3)	0.7 (0.03, 1.4)
BW, kg	Women	58.4 (57.9, 58.9)	58.2 (57.8, 58.7)	58.0 (57.6, 58.4)	59.1 (58.9, 59.4)	60.0 (59.8, 60.3)	59.8 (59.5, 60.1)	59.9 (59.6, 60.2)
Difference in BW, kg		Ref	–0.2 (–1.0, 0.6)	–0.4 (–1.2, 0.4)	0.7 (0.03, 1.4)	1.6 (1.0, 2.3)	1.4 (0.7, 2.1)	1.5 (0.8, 2.2)

*BMI, body mass index; BW, body weight.*

*P value for trend was tested by linear regression analyses after adjustment of age.*

*Data are expressed as means (95% confidence interval).*

### Secular Difference in the Prevalence of Body Mass Index Categories

The prevalence of underweight decreased only in men aged 70–79 years (*p* for trend = 0.02) while it was similar in other groups. The prevalence of overweight increased in both men and women aged 70–79 years (both *p* for trend < 0.05). The prevalence of obesity increased with natural year in men aged 70–79 years and all women except 65–69 years (all *p* for trend < 0.05) (Table 4).

**TABLE 4:** The prevalence of underweight, overweight and obesity in 50,192 Chinese aged participants from 2014 to 2020.

Sex	BMI category	Age	Year							P-trend
			2014	2015	2016	2017	2018	2019	2020	
Men (*n* = 25,505)	Underweight	65–69 y	1.5	1.2	1.3	1.0	1.0	1.3	0.7	0.12
		70–79 y	3.5	3.2	3.0	2.7	2.1	2.0	2.6	0.02
		≥ 80 y	4.9	4.5	5.4	5.6	4.9	5.5	5.1	0.39
	Overweight	65–69 y	45.1	42.2	43.0	43.4	45.1	45.3	41.8	0.87
		70–79 y	38.6	37.1	38.9	42.5	40.8	42.5	42.2	0.02
		≥ 80 y	32.4	31.8	29.7	29.0	35.6	35.8	40.3	0.06
	Obesity	65–69 y	4.7	4.8	5.1	5.6	6.4	5.1	6.3	0.06
		70–79 y	3.8	3.8	4.5	4.5	5.9	6.7	6.0	< 0.01
		≥ 80	3.7	4.7	4.2	4.7	3.2	6.4	5.8	0.16
Women (*n* = 24,687)	Underweight	65–69 y	2.8	1.8	1.9	1.8	2.3	1.7	1.6	0.12
		70–79 y	1.8	2.7	3.1	3.0	2.2	1.9	1.5	0.39
		≥ 80 y	5.3	6.1	3.7	5.3	3.4	4.6	4.0	0.18
	Overweight	65–69 y	38.3	36.4	34.3	38.5	39.3	38.4	38.3	0.37
		70–79 y	38.5	33.7	37.0	37.8	40.9	40.3	42.0	< 0.05
		≥ 80 y	36.0	27.9	33.6	34.4	35.1	36.9	38.7	0.14
	Obesity	65–69 y	6.7	7.2	5.0	8.3	7.5	7.6	9.0	0.13
		70–79 y	6.5	4.6	3.7	8.9	9.6	10.8	9.8	0.04
		≥ 80 y	3.3	4.1	4.1	7.5	9.6	8.8	6.6	< 0.05

*World Health Organization (WHO) criteria was used to classify participants into four groups: underweight (BMI < 18.5 kg/m^2^), normal weight (18.5 kg/m^2^ ≤ BMI < 25 kg/m^2^), overweight (25 kg/m^2^ ≤ BMI < 30 kg/m^2^), and obesity (BMI ≥ 30 kg/m^2^).*

*P-values for trends were determined by linear regression after setting year as the continuous variable.*

**FIGURE 1:**
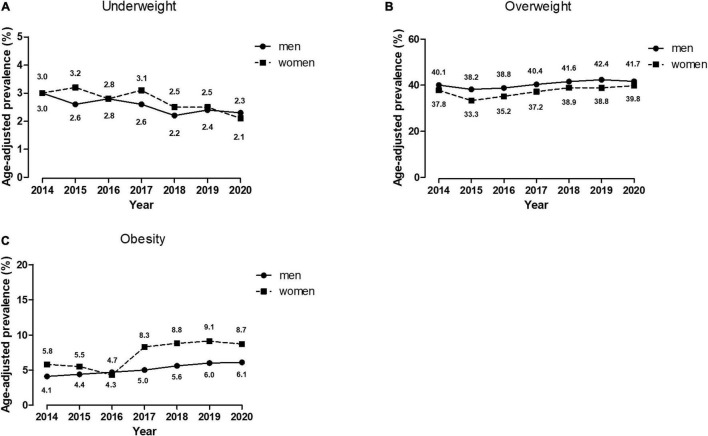
Age-standardized prevalence of underweight, overweight and obesity in 50,192 Chinese aged participants from 2014 to 2020. The estimated prevalence was age-standardized to the 2020 census of the Shanghai Aged population by the direct method. **(A)** Age-adjusted prevalence of underweight; **(B)** Age-adjusted prevalence of overweight; **(C)** Age-adjusted prevalence of obesity.

The age-standardized prevalence of underweight, overweight, and obesity from 2014 to 2020 were shown in Figure 1. From 2014 to 2020, age-standardized prevalence of underweight decreased from 3.0 to 2.3% in men and from 3.0 to 2.1% in women. Overweight was more prevalent in men than in women. Age-standardized prevalence of overweight increased in both men (from 40.1 to 41.7%) and women (from 37.8 to 39.8%) and so as obesity (men: from 4.1 to 6.1%; women: from 5.8 to 8.7%).

## Discussion

In the present study including 50,192 Chinese aged participants, we determined that the height, body weight, and BMI decreased with age. From 2014 to 2020, the age-standardized prevalence of underweight decreased while overweight and obesity slightly increased in both men and women.

We found that the height and body weight decreased with age in the current aged population. The English Longitudinal Study of Aging found that reduction in height is an important phenomenon among respondents aged 50 years and over. On average, physical stature decline occurs at an annual rate of between 0.08 and 0.10% for men, and 0.12 and 0.14% for women, which approximately translates into a 2–4 cm reduction in height over the life course (25). In addition to osteoporosis and certain kinds of arthritis, height shrinkage during aging is also associated with socioeconomic status (26). The similar age-related decline in weight was also observed several prospective studies (27–29). A longitudinal study also reported that BMI declined significantly among older adults. The difference in BMI was 0.435 kg/m^2^ in the younger-old (60–77 years) and was 3.48 kg/m^2^ in the older-old (≥ 78 years) during 15 years of follow up (30). Unintentional weight loss and associated adverse outcomes in older people may be attributable to protein-energy malnutrition, cachexia, the physiological anorexia of aging, or some combination of chronic diseases (31).

The similar downward trend in underweight in aged population was confirmed in both Chinese (22) (2013–2018) and Korean (17) (1998–2014) nationwide previous studies, but somewhat different from the Japanese aged population (2003–2016), in whom the prevalence of underweight decreased in men, but increased in women (32). In Brazil (33), Nepal (34), the Philippines, and Taiwan (35), the prevalence of underweight was higher in women. These studies were in line with our study.

Similar to our findings, some studies also reported increasing trend in overweight and obesity in both older men and women, such as a Iranian (36) study (2002–2014) and a Chinese (22) previous study (2013–2018). A Japanese study (2002–2016) was somewhat different, which reported that overweight and obesity increased in older men but decreased in older women (32). Conversely, the mean body weight of older Norwegians (65–79 years) decreased in both men and women from 2007 to 2016 (37). A previous study which such as Chinese elderly (60–69 years) reported annual changes in mean BMI (calculated as the absolute difference mean BMI between the start and end years divided by total number of years covered) from 2010 to 2018 was 0.08 kg/m^2^ in men, while 0.04 kg/m^2^ in women (21). Sex differences in growth rate were opposite to our findings that in Chinese adults (≥ 65 years), annual changes in the mean BMI from 2014 to 2020 was 0.05 kg/m^2^ in men, while 0.08 kg/m^2^ in women. The reason might lie on study period, age distribution, and cities where the participants were recruited. The increasing trend in overweight and obesity may be caused by increased consumption of animal food, soft beverage, and decreased physical activities (38, 39). Obesity is the result of disrupted energy balance, which is partially the consequence of alterations in the hypothalamic melanocortin circuitry. Although our understanding of energy management and the interactions between intake, metabolism, and expenditure are not yet fully understood (40), evidences suggested that unhealthy diet and lifestyles could impair the sophisticated hypothalamic circuits that regulate energy homeostasis, thus contributing to obesity (41). Unhealthy diet and lifestyle, such as less home cooking, more reliance on convenience food, frequent snacking, sweets, soft drinks, and fast food, sedentary behavior, and less physical activity, are important risk factors for obesity (42–44).

In 2017, China have released the 13th Five-Year Plan for Healthy Aging, the first national policy, to promote healthy living among the older population (45). This was an important landmark, signaling healthy aging as a key priority in the national political agenda for health (46). Furthermore, the China National Nutrition Plan 2017–2030, has included “senior nutrition improvement action” as one of the major initiatives of the country (47), which included regularly monitoring and evaluating nutritional status of the elderly, providing dietary guidance and consultation for the elderly, and carrying out special nutritional interventions for the elderly with low body weight. Our findings are helpful in dealing with nutrition problems for the older population and designing evidence-based policies to reduce both underweight and overweight/obesity in the Chinese aged population.

### Recommendations

Although further screening is needed to assess the longer-term trends and changing patterns, the present study findings highlight the pressing need for more targeted health policies to reduce further increases in obesity in Chinese aged population, with a gradual shift toward placing more emphasis on older women.

### Strengthens and Limitations

Our study has several strengths, such as a large sample size, and reliable measurements by trained staff in each survey. However, some limitations should also be considered. First, this was a cross-sectional study, which could not identify cause-and-effect relationships. Second, measurements of body fatness were not available. Thus, we cannot distinguish whether these differences resulted from body fat or muscle mass changes or both, which might underestimated the prevalence of muscle loss (48). Finally, other factors that may lead to an increased or decreased risk of underweight and overweight, such as socioeconomic status (7, 21), tobacco use (49), dietary habits, and physical activity were missing.

## Conclusion

Our results confirmed that BMI gradually increased from 2014 to 2020. The age-adjusted mean BMI increased by 0.3 kg/m^2^ in older men, and 0.5 kg/m^2^ in older women. The age- and sex-standardized prevalence of overweight and obesity significantly increased, especially in 70–79-year age group, while the prevalence of underweight decreased. The combination of a balanced-diet and physical exercise is needed to maintain optimal BMI range for aged population.

## Data Availability Statement

The raw data supporting the conclusions of this article will be made available by the authors, without undue reservation.

## Ethics Statement

The studies involving human participants were reviewed and approved by the Ethics Committee of Renji Hospital, School of Medicine, Shanghai Jiao Tong University. Written informed consent for participation was not required for this study in accordance with the national legislation and the institutional requirements.

## Author Contributions

YJ performed the analysis, interpreted the data, and drafted the manuscript. YJ, RX, XZ, ZC, and XG contributed to the conception and design of the study. WH contributed to the access of data. TX provided suggestions on the revision of statistical codes and supports on epidemiological insights. RX was the guarantor analyses of this work and had full access to all the data in the study and takes responsibility for the integrity of the data and the accuracy of the data analysis. All authors contributed to the critical revision and approval to submit the manuscript.

## Conflict of Interest

The authors declare that the research was conducted in the absence of any commercial or financial relationships that could be construed as a potential conflict of interest.

## Publisher’s Note

All claims expressed in this article are solely those of the authors and do not necessarily represent those of their affiliated organizations, or those of the publisher, the editors and the reviewers. Any product that may be evaluated in this article, or claim that may be made by its manufacturer, is not guaranteed or endorsed by the publisher.
