# Supplementary material for: Secular Difference in Body Mass Index From 2014 to 2020 in Chinese Older Adults: A Time-Series Cross-Sectional Study
Source: Front Nutr. 2022 Jun 21;9:923539. doi: 10.3389/fnut.2022.923539 (PMC9253615; doi:10.3389/fnut.2022.923539)

**Supplementary table 1. Age-specific trends in anthropometric data in 50,192 Chinese aged participants in each individual year**

| Year | Difference in variable | Men | | | P-trend | Women | | | P-trend |
| --- | --- | --- | --- | --- | --- | --- | --- | --- | --- |
|  |  | 65-69y | 70-79y | ≥80y |  | 65-69 | 70-79 | ≥80 |  |
| 2014 | Height, cm | Ref | -1.3 (-1.9, -0.7) | -2.4 (-3.1, -1.7) | < .01 | Ref | -1.7 (-2.4, -1.0) | -4.6 (-5.7, -3.5) | < .01 |
|  | BW, kg | Ref | -2.6 (-3.5, -1.6) | -5.0 (-6.1, -3.8) | < .01 | Ref | -1.5 (-2.6, -0.4) | -5.7 (-7.4, -3.9) | < .01 |
|  | BMI, kg/m^2^ | Ref | -0.5 (-0.8, -0.2) | -1.1 (-1.4, -0.7) | < .01 | Ref | -0.1 (-0.5, 0.3) | -0.9 (-1.6, -0.3) | .01 |
| 2015 | Height, cm | Ref | -1.2 (-1.8, -0.7) | -2.6 (-3.3, -1.9) | < .01 | Ref | -1.7 (-2.4, -1.0) | -4.3 (-5.5, -3.2) | < .01 |
|  | BW, kg | Ref | -2.3 (-3.2, -1.3) | -4.8 (-6.0, -3.7) | < .01 | Ref | -2.7 (-3.7, -1.6) | -5.5 (-7.3, -3.7) | < .01 |
|  | BMI, kg/m^2^ | Ref | -0.4 (-0.7, -0.2) | -1.0 (-1.3, -0.6) | < .01 | Ref | -0.6 (-1.0, -0.2) | -0.9 (-1.6, -0.3) | < .01 |
| 2016 | Height, cm | Ref | -1.4 (-2.0, -0.9) | -3.4 (-4.0, -2.7) | < .01 | Ref | -1.8 (-2.4, -1.2) | -4.2 (-5.2, -3.3) | < .01 |
|  | BW, kg | Ref | -2.4 (-3.2, -1.5) | -6.0 (-7.1, 5.0) | < .01 | Ref | -1.5 (-2.5, -0.6) | -3.9 (-5.3, -2.4) | < .01 |
|  | BMI, kg/m^2^ | Ref | -0.4 (-0.7, -0.2) | -1.2 (-1.5, -0.9) | < .01 | Ref | -0.1 (-0.4, 0.3) | -0.3 (-0.9, 0.2) | .24 |
| 2017 | Height, cm | Ref | -1.6 (-2.0, -1.2) | -2.9 (-3.5, -2.3) | < .01 | Ref | -2.3 (-2.7, -1.9) | -5.0 (-5.5, -4.5) | < .01 |
|  | BW, kg | Ref | -2.1 (-2.7, -1.4) | -5.8 (-6.8, -4.9) | < .01 | Ref | -1.9 (-2.5, -1.3) | -5.1 (-5.9, -4.2) | < .01 |
|  | BMI, kg/m^2^ | Ref | -0.3 (-0.5, -0.1) | -1.2 (-1.5, -0.9) | < .01 | Ref | -0.1 (-0.3, 0.2) | -0.5 (-0.8, -0.2) | < .01 |
| 2018 | Height, cm | Ref | -1.4 (-1.8, -0.9) | -2.5 (-3.2, -1.9) | < .01 | Ref | -2.6 (-3.0, -2.2) | -6.7 (-7.3, -6.1) | < .01 |
|  | BW, kg | Ref | -1.8 (-2.4, -1.2) | -5.4 (-6.3, -4.4) | < .01 | Ref | -1.6 (-2.2, -0.9) | -5.6 (-6.5, -4.7) | < .01 |
|  | BMI, kg/m^2^ | Ref | -0.2 (-0.4, -0.03) | -1.2 (-1.5, -0.9) | < .01 | Ref | 0.2 (-0.03, 0.4) | -0.2 (-0.5, 0.2) | .92 |
| 2019 | Height, cm | Ref | -1.6 (-2.1, -1.1) | -3.5 (-4.4, -2.5) | < .01 | Ref | -2.4 (-2.8, -2.0) | -5.4 (-6.0, -4.8) | < .01 |
|  | BW, kg | Ref | -1.5 (-2.4, -0.7) | -4.7 (-6.3, -3.1) | < .01 | Ref | -1.1 (-1.7, -0.4) | -4.8 (-5.7, -3.8) | < .01 |
|  | BMI, kg/m^2^ | Ref | -0.1 (-0.3, 0.2) | -0.7 (-1.2, -0.2) | .01 | Ref | 0.3 (0.1, 0.6) | -0.3 (-0.7, 0.1) | .75 |
| 2020 | Height, cm | Ref | -2.0 (-2.5, -1.5) | -4.1 (-5.3, -3.0) | < .01 | Ref | -2.2 (-2.6, -1.8) | -5.2 (-5.8, -4.6) | < .01 |
|  | BW, kg | Ref | -2.0 (-2.9, -1.1) | -4.8 (-6.8, -2.8) | < .01 | Ref | -1.2 (-1.9, -0.5) | -5.0 (-5.9, -4.0) | < .01 |
|  | BMI, kg/m^2^ | Ref | -0.1 (-0.4, 0.2) | -0.5 (-1.2, 0.1) | .08 | Ref | 0.2 (-0.04, 0.5) | -0.4 (-0.8, -0.1) | .18 |

**Note**:

1. Abbreviation: **BMI**, body mass index; **BW**, body weight.

2. Linear regression analyses were used to assess the trend in mean height, BW and BMI with age in each individual year.

3. Data are expressed as means (95% confidence interval).

**Supplementary table 2A. Trends in anthropometric parameters in different age groups in 25,505 Chinese aged men from 2014 to 2020.**

| Age group | Difference in variable | Year group | | | | | | | β coefficient | P-trend |
| --- | --- | --- | --- | --- | --- | --- | --- | --- | --- | --- |
|  |  | 2014 | 2015 | 2016 | 2017 | 2018 | 2019 | 2020 |  |  |
| 65-69 y | Height, cm | Ref | 0.6 (-0.05, 1.3) | 1.0 (0.4, 1.7) | 0.8 (0.3, 1.4) | -0.03 (-0.6, 0.5) | 0.1 (-0.5, 0.7) | 0.6 (-0.05, 1.2) | -.04 | .19 |
|  | BW, kg | Ref | 0.3 (-0.7, 1.4) | 0.9 (-0.1, 1.9) | 0.6 (-0.3, 1.5) | 0.4 (-0.5, 1.3) | 0.4 (-0.6, 1.4) | 0.7 (-0.3, 1.7) | .05 | .34 |
|  | BMI, kg/m^2^ | Ref | -0.1 (-0.4, 0.3) | 0.02 (-0.3, 0.3) | -0.02 (-0.3, 0.3) | 0.2 (-0.1, 0.4) | 0.1 (-0.2, 0.4) | 0.1 (-0.2, 0.4) | .03 | .07 |
| 70-79 y | Height, cm | Ref | 0.6 (-0.05, 1.3) | 0.8 (0.2, 1.5) | 0.4 (-0.2, 1.0) | -0.3 (-0.9, 0.3) | -0.4 (-1.1, 0.2) | -0.3 (-1.0, 0.3) | -.17 | < .01 |
|  | BW, kg | Ref | 0.6 (-0.5, 1.7) | 1.0 (-0.1, 2.1) | 1.0 (-0.01, 1.9) | 1.0 (0.002, 1.9) | 1.1 (0.1, 2.1) | 0.9 (-0.1, 2.0) | .13 | .02 |
|  | BMI, kg/m^2^ | Ref | 0.03 (-0.3, 0.4) | 0.1 (-0.2, 0.5) | 0.2 (-0.1, 0.5) | 0.4 (0.1, 0.7) | 0.5 (0.2, 0.9) | 0.4 (0.1, 0.8) | .10 | < .01 |
| ≥80 y | Height, cm | Ref | 0.4 (-0.5, 1.4) | 0.2 (-0.8, 1.1) | 0.4 (-0.5, 1.3) | -0.2 (-1.1, 0.7) | -1.2 (-2.4, 0.02) | -1.5 (-2.9, 0.05) | -.19 | < .01 |
|  | BW, kg | Ref | 0.5 (-1.1, 2.0) | -0.05 (-1.5, 1.4) | -0.1 (-1.6, 1.3) | 0.1 (-1.4, 1.6) | 0.4 (-1.6, 2.4) | 0.4 (-1.9, 2.8) | .02 | .85 |
|  | BMI, kg/m^2^ | Ref | 0.05 (-0.5, 0.6) | -0.1 (-0.6, 0.4) | -0.2 (-0.6, 0.3) | 0.1 (-0.4, 0.6) | 0.5 (-0.2, 1.1) | 0.6 (-0.2, 1.4) | .06 | .07 |

**Note:**

1. Abbreviation: **BMI**, body mass index; **BW**, body weight.

2. Linear regression analyses were used to assess the trend in mean height, BW, and BMI after adjustment of age.

3. Data are expressed as means (95% confidence interval).

**Supplementary table 2B. Trends in anthropometric parameters in different age groups in 24,687 Chinese aged women from 2014 to 2020.**

| Age group | Difference in variable | Year group | | | | | | | β coefficient | P-trend |
| --- | --- | --- | --- | --- | --- | --- | --- | --- | --- | --- |
|  |  | 2014 | 2015 | 2016 | 2017 | 2018 | 2019 | 2020 |  |  |
| 65-69 y | Height, cm | Ref | 0.6 (-0.2, 1.3) | 0.9 (0.2, 1.6) | 0.6 (0.01, 1.2) | 1.6 (1.0, 2.2) | 0.8 (0.2, 1.5) | 0.8 (0.2, 1.5) | .12 | < .01 |
|  | BW, kg | Ref | 0.3 (-0.9, 1.5) | -0.6 (-1.7, 0.6) | 0.7 (-0.3, 1.7) | 1.6 (0.6, 2.5) | 1.1 (0.1, 2.1) | 1.3 (0.2, 2.3) | .27 | < .01 |
|  | BMI, kg/m^2^ | Ref | -0.1 (-0.5, 0.4) | -0.5 (-0.9, -0.1) | 0.1 (-0.3, 0.5) | 0.1 (-0.2, 0.5) | 0.1 (-0.2,0.5) | 0.2 (-0.1, 0.6) | .08 | < .01 |
| 70-79 y | Height, cm | Ref | 0.7 (-0.1, 1.5) | 0.7 (-0.01, 1.5) | 0.04 (-0.6, 0.7) | 0.7 (0.1, 1.4) | 0.1 (-0.6, 0.7) | 0.2 (-0.4, 0.9) | -.02 | .48 |
|  | BW, kg | Ref | -0.8 (-2.1, 0.4) | -0.6 (-1.8, 0.6) | 0.4 (-0.6, 1.4) | 1.6 (0.6, 2.7) | 1.5 (0.4, 2.5) | 1.6 (0.5, 2.6) | .42 | < .01 |
|  | BMI, kg/m^2^ | Ref | -0.6 (-1.0, -0.1) | -0.5 (-1.0, -0.02) | 0.2 (-0.2, 0.6) | 0.5 (0.1, 0.9) | 0.6 (0.2, 1.0) | 0.6 (0.2, 1.0) | .19 | < .01 |
| ≥80 y | Height, cm | Ref | 0.8 (-1.0, 2.6) | 1.2 (-0.5, 2.8) | 0.3 (-1.1, 1.7) | -0.3 (-1.8, 1.1) | 0.2 (-1.2, 1.6) | 0.4 (-1.1, 1.8) | -.05 | .44 |
|  | BW, kg | Ref | 0.3 (-2.3, 3.0) | 1.2 (-1.3, 3.6) | 1.5 (-0.5, 3.5) | 1.8 (-0.2, 3.9) | 2.1 (0.1, 4.2) | 2.1 (0.003, 4.2) | .33 | < .01 |
|  | BMI, kg/m^2^ | Ref | -0.1 (-1.2, 1.0) | 0.1 (-0.9, 1.1) | 0.6 (-0.3, 1.4) | 0.9 (0.1, 1.8) | 0.9 (0.01, 1.7) | 0.8 (-0.1, 1.7) | .16 | < .01 |

**Note:**

1. Abbreviation: **BMI**, body mass index; **BW**, body weight.

2. Linear regression analyses were used to assess the trend in mean height, BW, and BMI.

3. Data are expressed as means (95% confidence interval).

**Supplementary Figure 1. Flow chart of sample recruitment.**


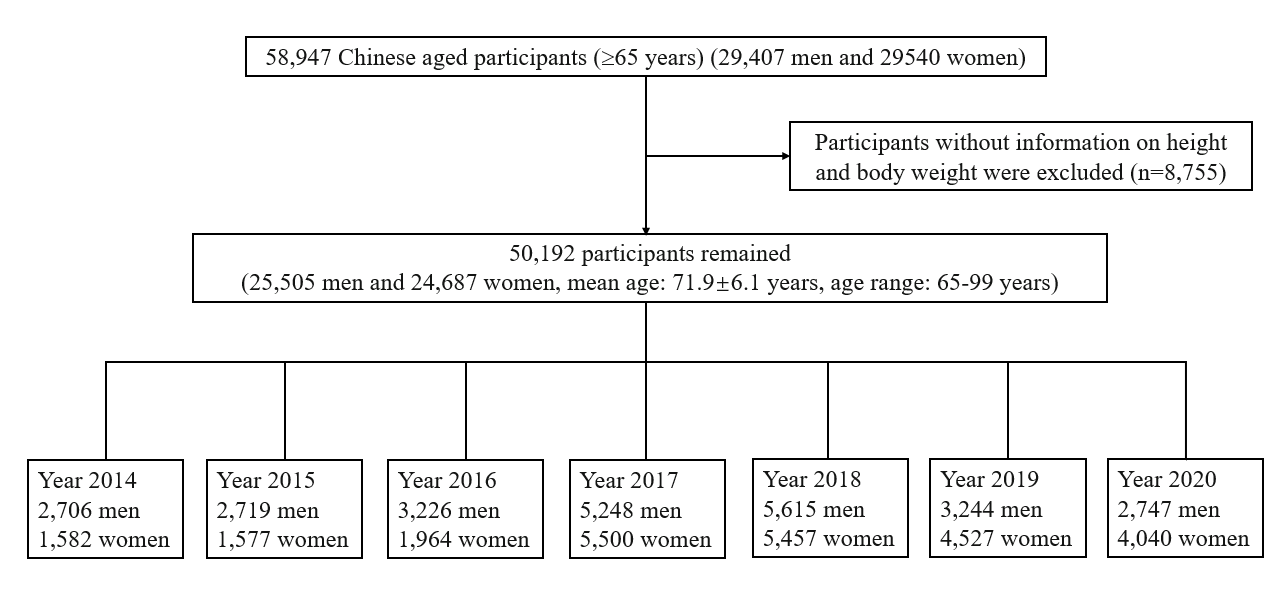

Supplement: Supplementary file 1 [file Data_Sheet_1.docx]
